# Investigation of Setting Time and Microstructural and Mechanical Properties of MK/GGBFS-Blended Geopolymer Pastes

**DOI:** 10.3390/ma15238431

**Published:** 2022-11-26

**Authors:** Qingyu Zhong, Xiang Tian, Guolun Xie, Xi Luo, Hui Peng

**Affiliations:** School of Civil Engineering, Changsha University of Science and Technology, Changsha 410114, China

**Keywords:** MK/GGBFS, geopolymer, isothermal calorimetry, pore structure, reaction extent

## Abstract

In this study, geopolymer pastes with 60% metakaolin (MK) and 40% ground granulated blast-furnace slag (GGBFS) were synthesized. To determine the influence of the alkaline activator concentration, modulus, and the liquid/solid (L/S) ratio on setting time and compressive strength, the geopolymerization process and microstructures of MK/GGBFS-blended geopolymer pastes were analyzed using isothermal calorimetry, X-ray diffraction, mercury intrusion porosimetry, and scanning electron microscopy. Acid dissolution was employed to measure reaction extent. The results showed that the initial setting time of the geopolymer pastes was between 68 and 226 min, and the initial setting and final setting time was apart about by 10 min. For the same variable, the total heat released was positively correlated to the reaction extent. Available silicate content increased the reaction rate and intensity at the initial stage, whereas the OH^−^ concentration controlled the reaction extent in the long term. A limited reaction extent existed in the geopolymeric reaction even if the system contained sufficient alkali content and medium. An increase in the L/S ratio increased the reaction extent. The highest reaction extent of 86.3% was found in the study. Additionally, increasing the L/S ratio reduced the compressive strength by increasing the porosity.

## 1. Introduction

Geopolymers are cementitious materials with three-dimensional networks configured by silicon–oxygen and aluminum–oxygen tetrahedrons [1]. Although Davidovits claimed that geopolymers differ from alkali-activated materials (AAMs) in terms of their chemistry, molecular structure, and long-term durability, several studies have used the term “geopolymer” for AAMs, even in high-calcium alkali-activated systems [2]. In recent decades, geopolymers have garnered increasing research interest because they exhibit excellent mechanical properties [3], high heat resistance [4], and high corrosion resistance [5,6]. Furthermore, geopolymeric reactions usually employ industrial waste, such as fly ash [7] and ground granulated blast-furnace slag (GGBFS) [8], mine tailings [9], and construction and demolition waste (CDW) [10] as precursors, reducing the environmental impact of the CO_2_ produced during the preparation of ordinary Portland cement (OPC) [11]. Producing 1 m^3^ of geopolymer with a compressive strength of 25 MPa produces 40% less CO_2_ emissions than producing the same quantity of OPC. This reduction further increases with increasing compressive strength design [12]. Therefore, geopolymers are potential cementitious materials for the development of green alternatives to OPC.

Metakaolin (MK) is obtained by heating kaolin at high temperature ranged from 550 to 900 °C. It is of good pozzolanic properties and can be used as a raw material for the synthesis of geopolymers [13]. However, metakaolin-based geopolymers are slow to harden at room temperature [14]. Additionally, MK has a large specific surface area [15], for which it requires a large amount of water during the preparation process and thus causing drying shrinkage and cracking problems during curing [16]. It has been shown that the content of oxide composition and of aluminosilicate raw materials have a significant effect on the mechanical properties and durability of geopolymers [17]. Incorporating calcium-containing aluminosilicate materials such as GGBFS into MK as raw materials, the-blended geopolymers have better strength than the geopolymers made of MK only or GGBFS only [18].

In general, the mass ratio of MK and GGBFS, and the nature and dosage of alkaline activators, are the dominant factors affecting the properties of the geopolymer. Some researchers studied the influence of the mass ratio of MK and GGBFS on the setting time and compressive strength of geopolymers. Due to the low length to diameter ratio and high reactivity of GGBFS, the incorporation of GGBFS into MK to prepare geopolymer can accelerate the hardening rate at room temperature. Huseien et al. [19] and Bernal [20] found that the setting time of MK/GGBFS-blended geopolymers was shortened with the increase in GGBFS content. Yip et al. [21] and Peng et al. [22] found that the compressive strength of MK/GGBFS-blended geopolymer first increased and then decreased as the GGBFS mass fraction increased from 0 to 100%, and the optimum mass ratio of MK to GGBFS was 3:2 to reach the maximum compressive strength. Furthermore, an appropriate amount of GGBFS incorporation into the MK could induce the coexistence of C-(A)-S-H and N-A-S-H networks in-blended geopolymer [23,24]. The C-(A)-S-H network fills the pores in the N-A-S-H network and bridges the gaps between the different reaction products and unreacted particles, which resulted in a stable and dense microstructure with enhanced mechanical strength [25,26]. However, when increasing the content of GGBFS while keeping the unchanged L/S ratio, the difference in water demand between GGBFS and MK led to a relatively more water in the system. The excess water evaporates and leaves pores in the hardened paste, resulting in cracking. Moreover, the excessive amount of GGBFS resulted in more Ca^2+^ reacting with OH^−^ in the system to form calcium hydroxide precipitation. Calcium hydroxide further reacts with CO_2_ in the atmosphere to form calcium carbonate, deteriorating the compressive strength [27]. Based on the above analysis, the mass fractions of MK and GGBFS were fixed at 60 wt. % and 40 wt. %, respectively.

In terms of the effect of the character and amount of alkaline activators on the macroscopic properties of geopolymer pastes, Khalil et al. [28] studied the effects of the L/S ratio and the alkaline activator modulus on the compressive strength of MK/GGBFS-blended geopolymer with a GGBFS content of 50%. The increase in modulus from 1.1 to 1.7 or the increase in the L/S ratio from 0.5 to 0.7 decreased compressive strength. When studying the MK/GGBFS geopolymer-blended paste with the same GGBFS content, Burciaga-Díaz et al. [29] found that when the modulus changes, the compressive strength of the paste with different alkali content changes differently. Hasnaoui et al. [30] believed that the change of modulus had little effect on the setting time of MK/GGBFS-blended geopolymer, while Huseien et al. [19] pointed out that the increase in modulus prolongs the setting time of MK/GGBFS-blended geopolymer. Although Bernal [20] studied the effect of alkali content on the compressive strength, the modulus was also changed when the alkali content was changed in the mixing ratio design. Therefore, it is unable to confirm which factor was responsible. As far as the nature and dosage of alkaline activator are concerned, each of the above research works does not cover the contents of all components of alkaline activator (silicon content, alkali content, and water content) and the total amount of alkaline activator on the setting and hardening properties of MK/GGBFS-blended geopolymer. Furthermore, the relationship between the mechanical properties, reaction extent, and pore structures of different formulations is still not well documented.

Therefore, in this work, the geopolymerization process and microstructure of MK/GGBFS geopolymer-blended paste with different alkaline activator concentrations, moduli, and L/S ratios were analyzed by isothermal calorimetry, X-ray diffraction (XRD), mercury intrusion pore size measurement (MIP), and scanning electron microscopy (SEM). Acid dissolution was used to measure reaction extents. On this basis, the setting time and compressive strength of MK/GGBFS-blended geopolymer paste were measured, and we aimed to fully reveal the character and amount of alkaline activators on the setting and hardening properties of MK/GGBFS-blended geopolymer paste as well as the relationship between the setting time and the exothermic characteristics of geopolymerization, the compressive strength and the reaction extent, and the pore structure characteristics. 

## 2. Materials and Methods

### 2.1. Materials

MK has a specific surface area of 1.27 m^2^/g, and a specific gravity of 2700 kg/m^3^. GGBFS has a specific surface area of 1.11 m^2^/g, and a specific gravity of 2944 kg/m^3^. The size distributions of MK and GGBFS were measured using a laser diffraction analyzer, in which d10, d50, and d85 were 0.52, 0.82, and 3.74 µm for MK and 1.39, 4.45, and 10.55 µm for GGBFS, respectively. Table 1 lists the main chemical components of MK and GGBFS. The alkaline activator was composed of sodium hydroxide pellets and water glass. Sodium hydroxide is a chemically pure reagent (96%). Water glass is an industrial agent with a SiO_2_/Na_2_O ratio of 3.28, in which the mass ratios of SiO_2_, Na_2_O, and H_2_O were 26.54, 8.35, and 65.11%, respectively. 

### 2.2. Paste Synthesis

Table 2 lists the synthesis formulations of the MK/GGBFS-blended geopolymer pastes. The alkaline activator was prepared by mixing distilled water, NaOH pellets, and water glass and stirring for 4 h with a stirrer. The alkaline activator was then mixed with raw materials containing MK and GGBFS at a 60/40 mass ratio. The mixture was then poured into a cement mortar mixer and stirred for 5 min, slowly stirred for 2 min, followed by a break of 10 s, and then rapidly stirred for 3 min. The mixture was poured into a cubic mold (70.7 × 70.7 × 70.7 mm^3^) for curing at ambient temperature for 28 d. Abbreviations of “C-x,” “M-y,” and “L/S-z” were designed for pastes synthesized with variables of concentration, modulus, and L/S, respectively. x, y, and z denote the corresponding values.

### 2.3. Characterizations

Isothermal calorimetry (Tam Air) was performed to assess the hydration reaction process of pates at 25 °C for 72 h, which was manufactured by Wosite Co., Ltd. (Reston, VA, USA). Acid dissolution was conducted to measure the reaction extent based on a previous study [31]. The reference [31] shows that the dissolution rate of the reaction product in the MK geopolymer is 100% in hydrochloric acid with pH = 0, and the acid soluble residue is unreacted MK. Lecomte et al. [32] pointed out that GGBFS could completely react with sodium silicate in the alkaline activator when GGBFS geopolymer was cured for 28 days. We have also confirmed by XRD test that both MK geopolymer and MK/GGBFS-blended geopolymer with 20% to 80% GGBFS content have the same chemical composition as MK after acid dissolution; the dissolution rate of GGBFS geopolymer in the hydrochloric acid at pH = 0 is close to 100%. The process of acid dissolution test is as follows: the samples cured for 28 days were ground into a powder with a size < 74 μm and dried in an oven at 80 °C to a constant weight; 2 g powder (m_1_) was used for the acid dissolving process at pH = 0 with the continuous addition of hydrochloric acid. After dissolution was complete, the residue was filtered and washed with deionized water until the filtrate became transparent. The residue was dried and weighed (m_2_). Thus, the reaction extent (m_1_ − m_2_)/m_1_ was obtained. D8 Advance X-ray diffractometer was used to study the mineralogical crystals with Cu Kα radiation at 2θ between 10 and 80°, which was manufactured by Bruker Co., Ltd. (Bremen, Germany). Phenom ProX G6 desktop scanning electron microscopy was used to evaluate the morphological characteristics of the samples, which was manufactured by Phenom World (The Netherlands). PoreMaster-33GT series continuous scanning automatic mercury intrusion porosity analyzer was used to determine the pore distribution of the pastes, which was manufactured by Quantachrome Co., Ltd. (Boynton Beach, FL, USA). The initial and final setting times were measured using the Vicat method, based on GB/T1346. The compressive strength of the pastes was measured, and the resulting compressive strength was obtained by averaging the values of three samples in each group.

## 3. Results and Discussion

### 3.1. Isothermal Calorimetry

**Figure 1:**
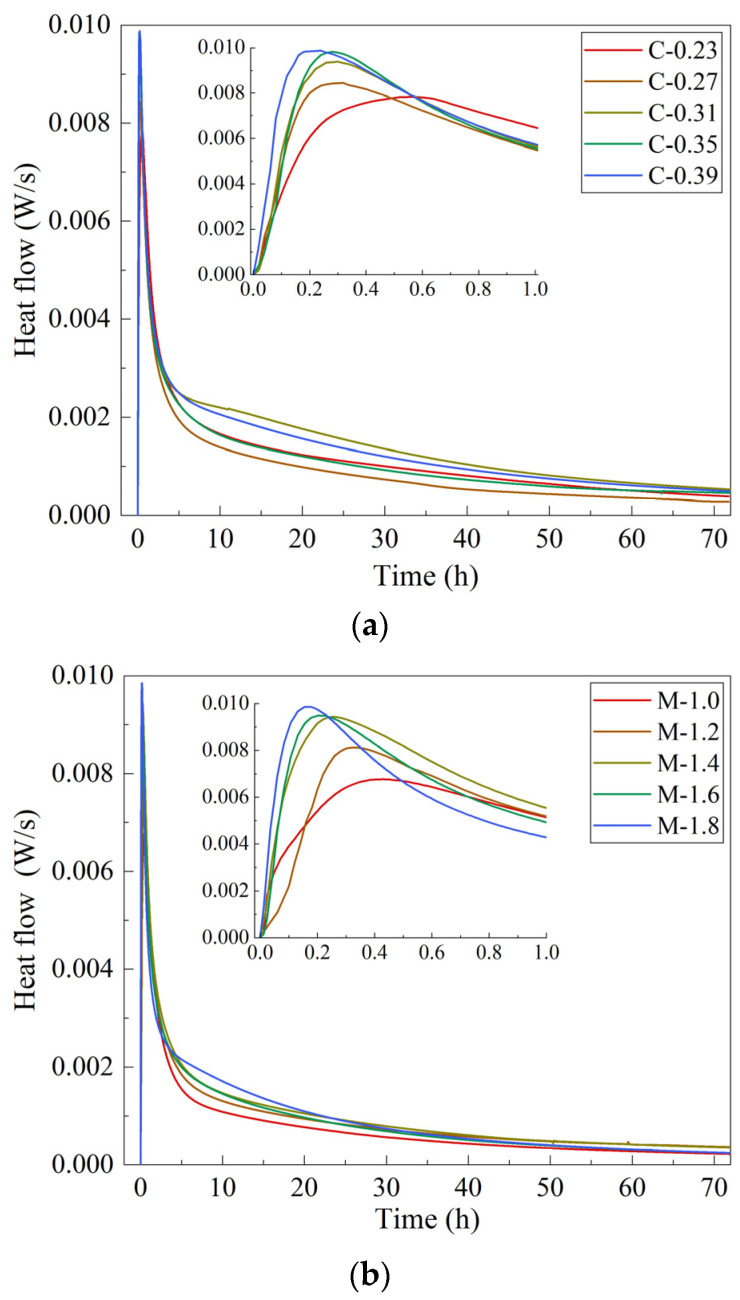

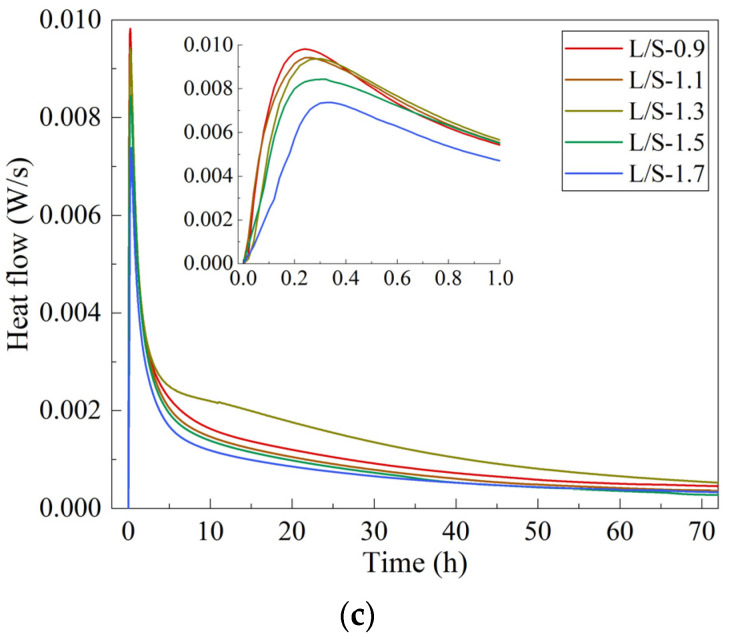
Heat evolution curves of MK-GGBFS geopolymer synthesized with varying alkaline activator concentrations (**a**), moduli (**b**), and L/S ratios (**c**).

Figure 1 and Table 3 show the heat-evolution curves and total heat release of the MK/GGBFS-blended geopolymer pastes synthesized with varying alkaline activator concentrations, moduli, and L/S ratios. The pastes exhibited one exothermic peak for all formulations. This might be attributed to the MK/GGBFS-blended system with high alkaline activator concentration. The geopolymerization in MK-based geopolymers typically exhibits two exothermic peaks [33]. The first one arises from the dissolution of MK when it comes into contact with an alkaline activator; when dissolved silicate and aluminate oligomers are concentrated, polycondensation begins and the second exothermic peak occurs [34]. In alkali-activated slag-based pastes, the number of exothermic peaks in the alkaline activation reaction depends on the alkaline concentration [35]. At low concentrations, dissolution and polycondensation occur separately because of the slow dissolution of raw materials, and an induction period is required between the dissolution and polycondensation stages. With increasing concentration, these two stages occur rapidly and simultaneously [36]. With a high concentration of alkaline activator, the MK/GGBFS-blended system followed the exothermic behavior of alkali-activated slag-based pastes in which the two peaks merged.

The geopolymerization in the geopolymer slurry usually presents two [33,37] or three [38] exothermic peaks. The first exothermic peak is caused by the depolymerization of aluminosilicate particles after the alkaline activator contacts the surface of the aluminosilicate materials; when the silicon monomer and aluminum monomer produced by depolymerization reach a certain concentration, they begin to condense to form aluminosilicate oligomers, and the second exothermic peak appears [34]. The appearance of the third exothermic peak is otherwise related to the stability of the microstructure of the reaction products [38]. The number of exothermic peaks that occur during the geopolymerization is largely dependent on the alkaline activator [35]. The lower concentration leads to a slower depolymerization rate of mineral raw materials. There is an induction period between the depolymerization stage and the polycondensation stage, and the polycondensation exothermic peak appears after the depolymerization exothermic peak. With the increase in alkali content, these two reaction stages may occur simultaneously, and the two exothermic peaks are combined [36]. Therefore, the reaction exothermic curve in the alkali-activated MK/GGBFS-blended system has only one exothermic peak, which is caused by the use of alkaline activator with high alkali content in the pastes.

With an increase in both the concentration and modulus, exothermic peaks occurred earlier and became more intense, indicating an increase in the reaction rate and intensity. An alkaline activator with a higher concentration favors the dissolution of the precursors more strongly [35]; therefore, the required concentration of monomer silicate and aluminate for the polycondensation reaction is reached faster than that at low concentrations, and the alkaline activator with a higher modulus, even if it has a lower OH^−^ concentration, provides more available silicate for the geopolymeric reaction in the initial stage, thus accelerating and intensifying the reaction. Table 4 shows, however, that the modulus increase from 1 to 1.2 favored heat release and then weakened it with the continuous increase in modulus from 1.2 to 1.8. This may be attributed to the excess OH^−^ concentration in the alkaline activator with a modulus of 1.0. As shown in Table 2, matrix M-1.0 contained the highest NaOH concentration, which hindered the reaction because the rapidly formed gel attached to the MK/GGBFS particles, suppressing further dissolution [39]. Thus, less heat was released from the system. In the moderate range, a high OH^−^ concentration favored the total heat release of the reaction [40].

The increase in the L/S ratio delayed the exothermic peaks and weakened the intensity but increased the total heat released. A high L/S ratio provided more OH^−^ and available silicate, favoring the reaction and heat release. Simultaneously, the liquid could serve as a thermal container to absorb the heat released from the hydration reaction; then, the heat discharged from the liquid was measured by the detector. This process postponed the detection time of heat release, causing smooth exothermic linearity at a high L/S ratio.

### 3.2. Reaction Extent

**Figure 2:**
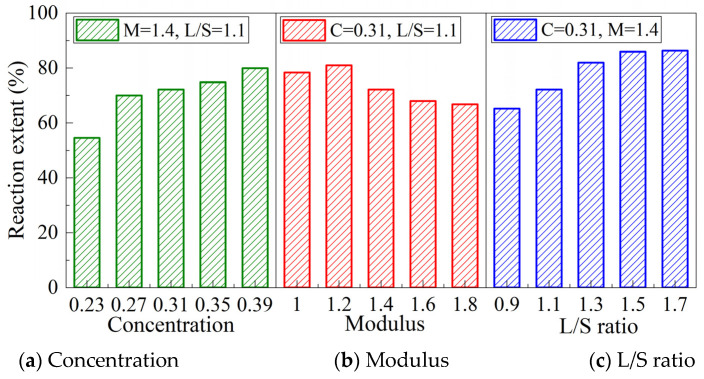
Reaction extent of MK-GGBFS geopolymer pastes with varying concentrations (**a**), moduli (**b**), and L/S ratios (**c**).

Figure 2 shows the reaction extent of MK/GGBFS-blended geopolymer pastes synthesized with varying concentrations, moduli of alkali activator, and L/S ratio. It can be seen from the diagram that when the modulus is 1.4 and the L/S ratio is 1.3, the reaction extent of the geopolymer paste increases with the increase in the concentration. The reaction extent of the paste with a concentration of 0.39 is 46.3% higher than that of the paste with a concentration of 0.23. When the modulus increased from 1.0 to 1.8, the reaction extent of the geopolymer paste increased first and then decreased and reached the maximum when the modulus was 1.2. When the other two factors are the same, the reaction extent of the paste increases by 32.5% when the L/S ratio increases from 0.9 to 1.7, and the reaction extent increases with the increase in the L/S ratio. 

As mentioned in Section 3.1, the exothermic peaks occur earlier and more intense at a higher modulus (Figure 1b) while exhibiting a lower reaction extent. This indicates that the available silicate promoted the reaction rate and intensity in the initial stage, whereas the OH^−^ concentration controlled the reaction extent in the long term. This is consistent with Reddy’s study [41] that increasing the SiO_2_ content in the alkaline activator accelerates the early reaction and the initial silicate hydrates formed in the system. The use of high pH alkaline activator is beneficial to the dissolution and polycondensation of the mineral raw materials and increases the amount of reaction products.

**Figure 3:**
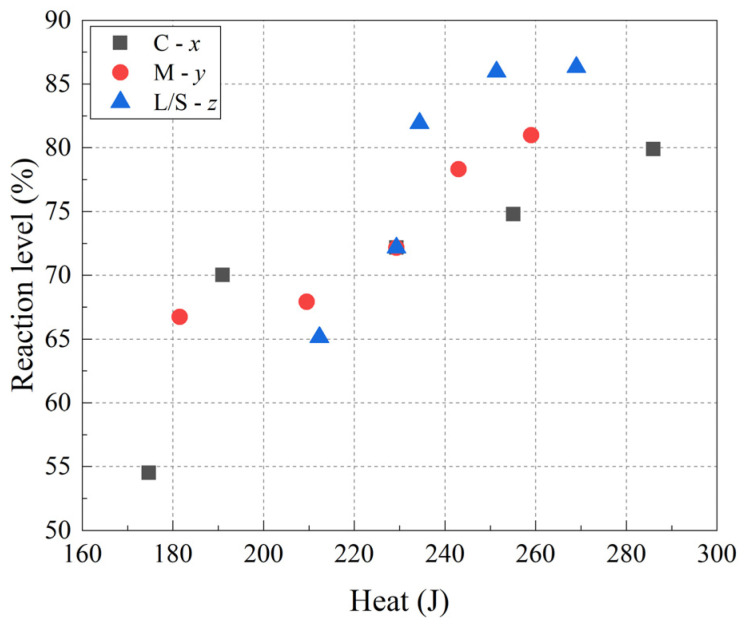
Reaction extent as a function of total heat released.

It is worth noting that the reaction extent increased from 65.1% to 81.9% with an increase in the L/S ratio from 0.9 to 1.3. However, when the L/S ratio was increased from 1.5 to 1.7, the reaction extent increased by only 0.4%. This phenomenon can be explained that water plays an mediating role in the geopolymerization [42]. With the increase in L/S ratio, water content and alkali content are conducive to the geopolymerization so the reaction extent increased rapidly. However, MK itself contains a small amount of quartz, and it is difficult for quartz to participate in the geopolymerization [43]. In addition, the product formed by the polycondensation reaction adheres to the surface of the unreacted mineral raw material, hindering the continuous generation of the precursor. Therefore, even if the reaction system contains sufficient alkali content and medium, a complete reaction is rarely achieved. There is a limit reaction extent in the alkali-activated MK-GGBFS composite system, and the limit reaction extent under the experimental conditions in this paper is 86.3%. Combined with the total heat released (Table 3), a relationship between the reaction extent and total heat released can be concluded, as shown in Figure 3. It can be seen from the figure that the paste with larger heat production does not necessarily have a higher reaction extent; for example, the heat production of sample C-0.39 and sample L-1.7 are 286 J and 269 J, respectively, and their reaction extents are 79.9% and 86.3%, respectively. This is because the degree of depolymerization of mineral raw materials determines the reaction extent, and the heat production is affected by the geopolymerization. The geopolymerization is not only related to the depolymerization degree of the mineral raw materials but also to the amount of effective silicate in the alkaline activator. When the number of effective silicates in the paste is different, there is no positive correlation between heat production and reaction extent.

### 3.3. Phase Composition

**Figure 4:**
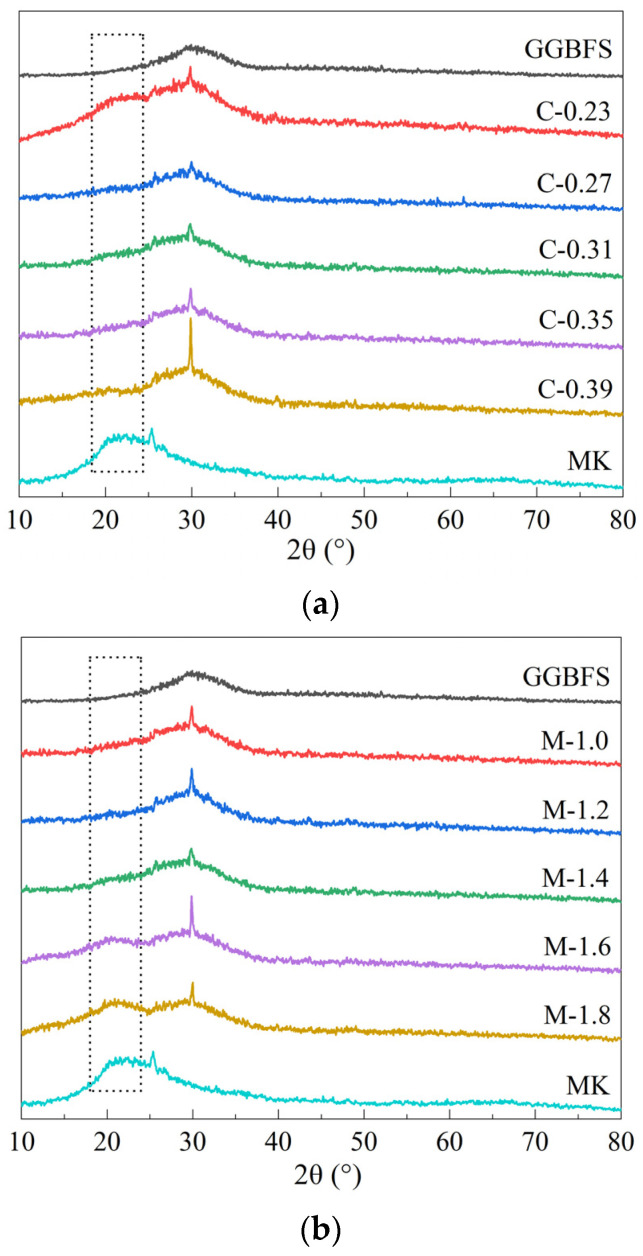

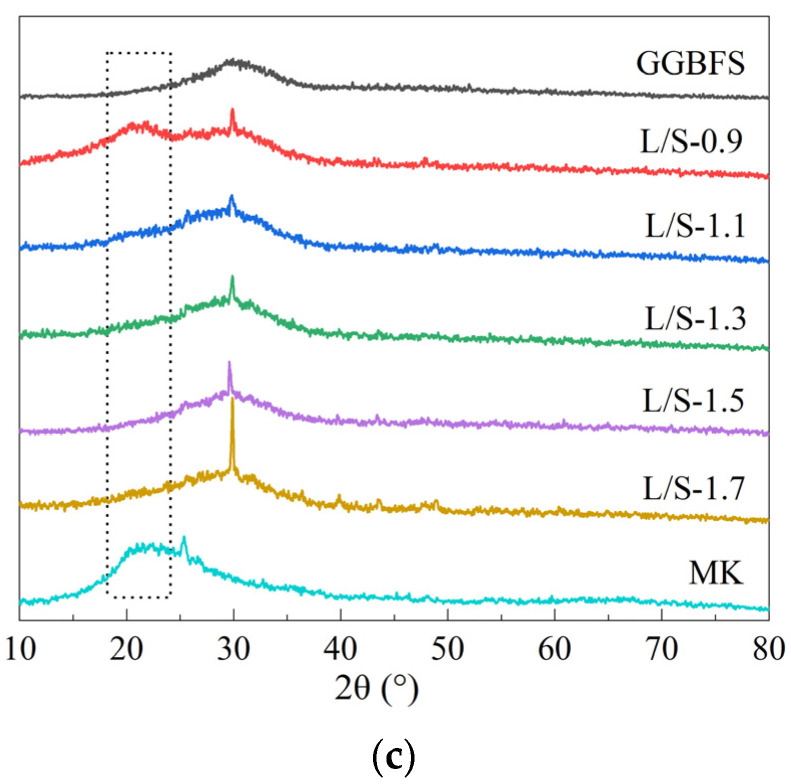
XRD patterns of samples under varying concentrations (**a**), moduli (**b**), and L/S ratio (**c**).

Figure 4 shows the XRD patterns of the MK/GGBFS-blended geopolymer pastes synthesized with different concentrations, moduli, and L/S ratios. MK and GGBFS exhibited amorphous peaks at 2θ values of 15–35° and 25–35°, respectively. Additionally, a small amount of quartz was present in MK, as found in previous studies [28]. After the geopolymeric reaction, a newly formed phase at 2θ of 29.3°, representing the C-(A)-S-H network [17,43], occurred in all the formulations. At a low concentration (0.23), an amorphous peak for MK was observed in the geopolymer. With an increase in concentration, the peak for MK disappeared [44]. In addition, the amorphous peak for MK existed at a high modulus (1.6 and 1.8) and low L/S ratio (0.9). This result is consistent with the reaction extent, in which a high concentration, low moduli, and high L/S ratio facilitate the extent of reaction. GGBFS exhibited a higher dissolution rate than MK because the former has a depolymerized siliceous structure and the latter has a reticulate structure. The shortage of alkali content at low concentration, high modulus, and high L/S ratio failed to dissolve all MK. In addition, MK remains in matrices with a low L/S ratio, which may be attributed to the insufficient medium to transfer the alkali to contact the MK particles.

### 3.4. Pore Structure

**Figure 5:**
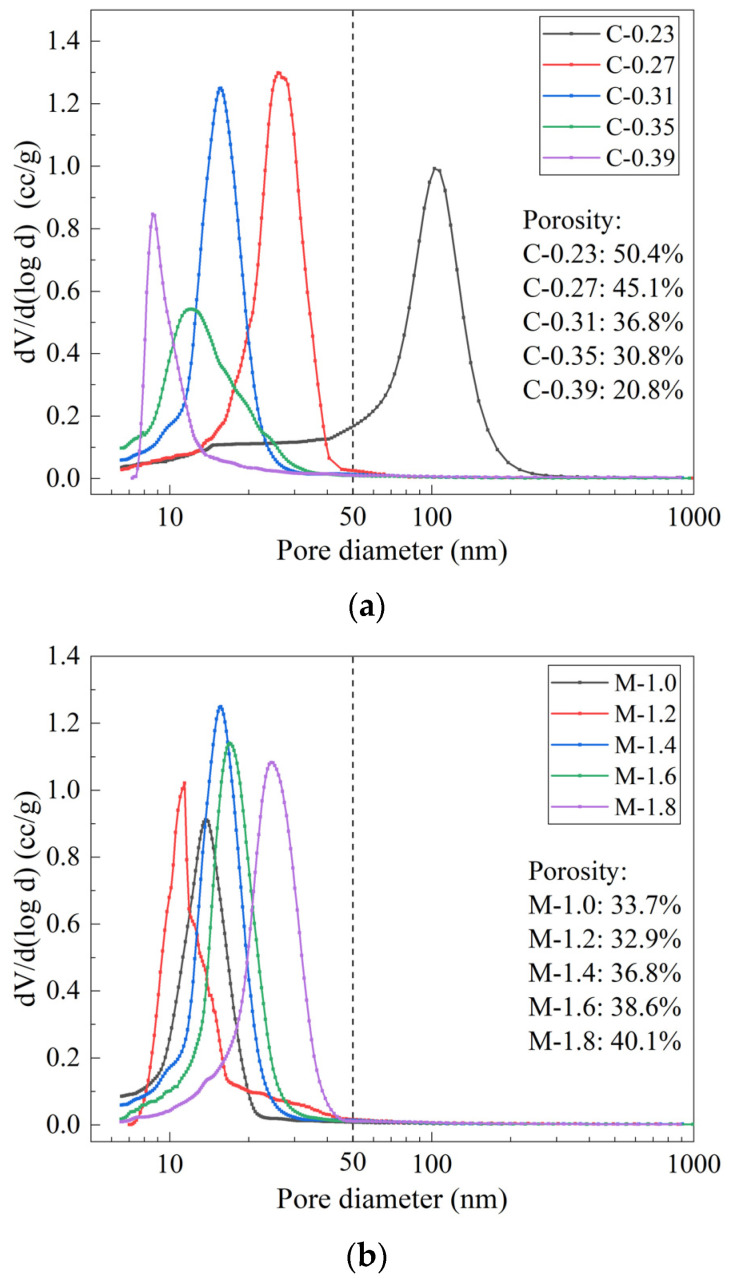

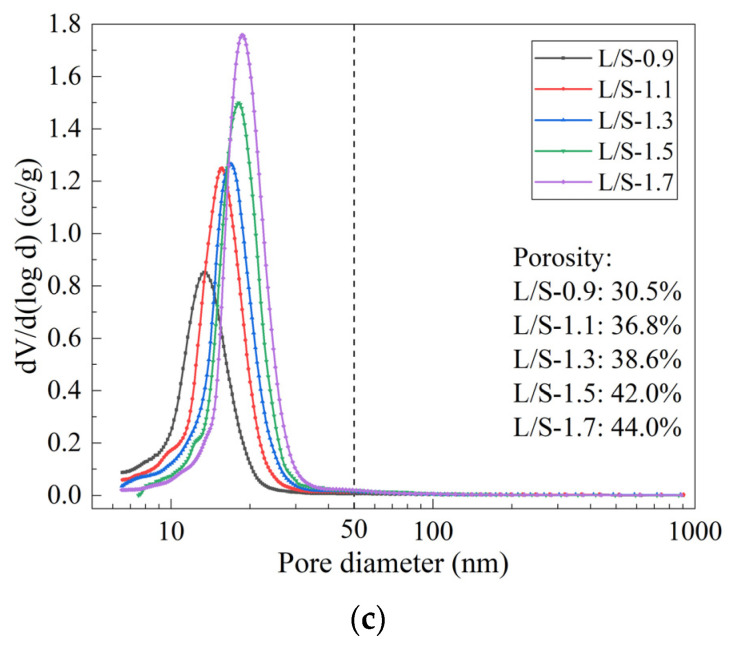
Pore distribution and porosity of MK/GGBFS-blended geopolymer pastes synthesized with varying concentrations (**a**), moduli (**b**), and L/S ratio (**c**).

Figure 5 shows the pore distribution and porosity of the MK/GGBFS-blended geopolymer pastes synthesized with varying concentrations, moduli of the alkaline activator, and L/S ratio. As the concentration increased, both pore size and porosity decreased. As the modulus increased from 1.0 to 1.2, both the pore size and porosity decreased slightly and then increased consistently from 1.2 to 1.8. However, with an increase in the L/S ratio, both the pore size and porosity rapidly increased. 

The pores of the geopolymer pastes are divided into four categories according to the pore size:gel pores (1000 nm), transition pores (10–100 nm), capillaries (100–1000 nm), and macropores (>1000 nm). The most probable pore size and the porosity of the pastes are shown in Table 4. The pore size corresponding to the peak in the pore structure curve of the paste is the most probable pore size. Except for samples C-0.23 and C-0.39, the most probable pore diameters of the other pastes are in the range of 11~26 nm. The smaller the total porosity, the smaller the most probable pore diameter. It can be seen from the table that the change of the three influencing factors leads to the change of the total porosity of the geopolymer paste mainly due to the change of the number of gel pores and transition pores. Except for the sample C-0.23, the numbers of capillary pores and macropores in the other samples are relatively low and do not change regularly. Capillary pores refer to the spaces that are not filled by the reaction product, and the fraction of pores in the MK/GGBFS-blended geopolymer paste is similar to the fraction of pores in the cement paste [45]. The macropores are mainly pores or defects, and their content has little to do with the influencing factors. This non-regular change may be due to the changes in the vibration molding process of different specimens and the random selection of MIP samples.

The larger the number of gels, the higher the strength of cement-based materials and the better the impermeability [46]. Studies have confirmed that the gel in the geopolymer paste is composed of gel particles with a diameter of 5~10 nm, and pores with a diameter of 2~50 nm are formed between the particles [47]. It can be seen that the gel formed is closely combined with the unreacted mineral raw material particles, and the pore structure is mainly gel pores.

**Figure 6:**
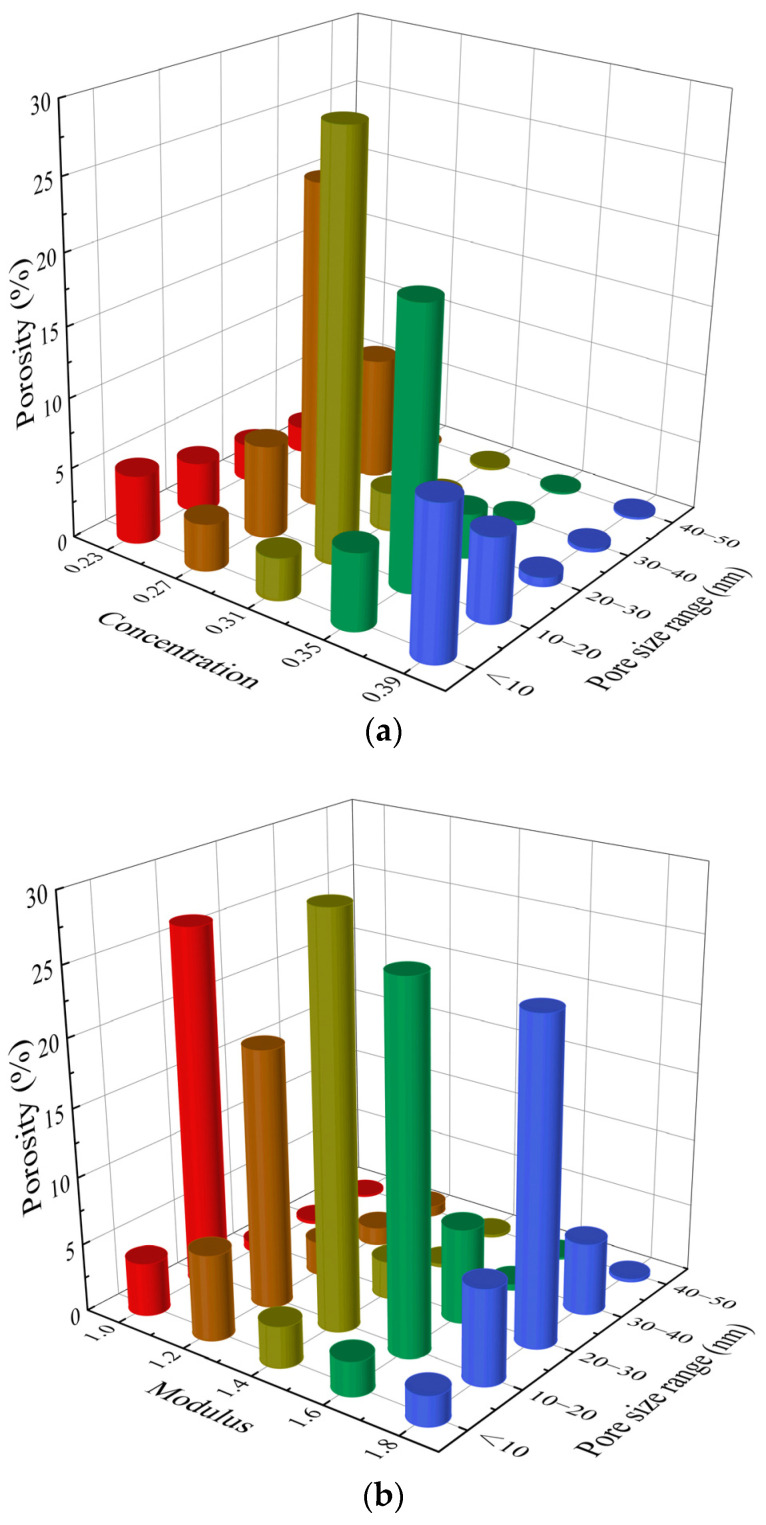

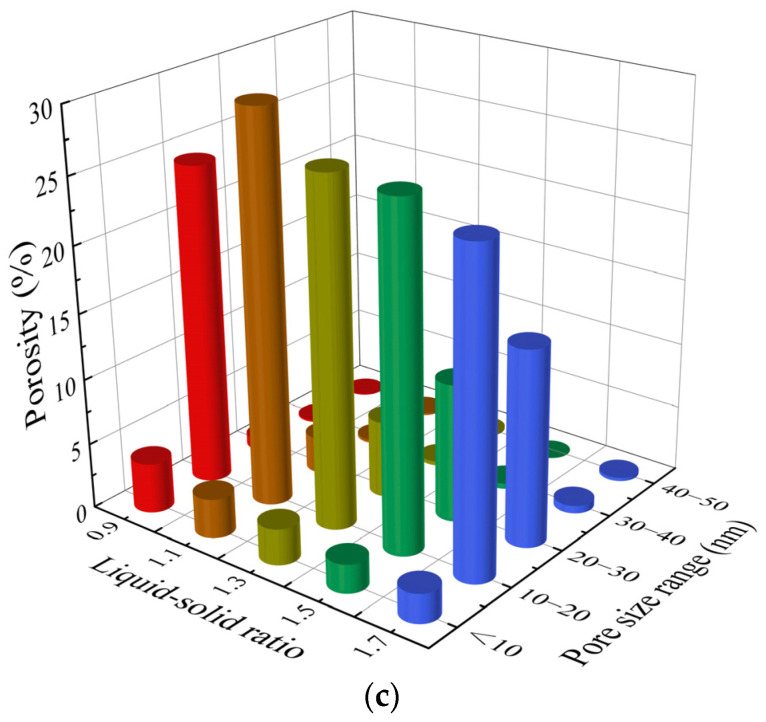
Porosity of pores smaller than 50 nm: concentrations (**a**), moduli (**b**), and L/S ratios (**c**).

In order to study the influence of the change of various influencing factors on the pore structure of the MK-GGBFS geopolymer paste, the porosity with a pore size of less than 50 nm is further subdivided, as shown in Figure 6. When the concentration increased from 0.23 to 0.27, the proportion of pores in the range of 20~30 nm increased significantly; when the concentration increased from 0.31 to 0.39, the proportion of pores with pore size less than 10 nm increased by 4.4 times. This indicates that increasing the solute concentration in the alkaline activator can promote the polycondensation reaction to generate more products to fill the space originally occupied by the raw materials and further refine the pores between the particles [48]. Ryu et al. [49] and Zheng et al. [50] also found that with the increase in alkali content, the pores in the fly ash geopolymer paste gradually dominated by mesopores (2~50 nm) and micropores (<2 nm). The proportion of pores with diameter less than 10 nm in the sample M-1.2 is obviously higher than that of other samples with different modulus. This result is consistent with the reaction extent. The high reaction extent indicates that more products was generated in the pastes, which filled the inner pores and interface between the particles [51].

When the modulus and concentration are constant, the L/S ratio increases from 0.9 to 1.7, and the pores less than 10 nm in the paste change little, but the porosity of the pores in the range of 10~20 nm decreases by 31.4%, and the porosity of the pores in the range of 20~30 nm increases by 10.7 times. This is because the effect of high L/S ratio on the pore structure characteristics of the MK-GGBFS geopolymer paste has two sides. On the one hand, the high L/S ratio leads to more water content in the system, which can provide more space for the depolymerization reaction of the mineral raw materials. The increase in alkali content and soluble silicate content can also promote the depolymerization reaction and provide more precursors for the polycondensation reaction. Therefore, the number of reaction products increases and the reaction extent increases. This conclusion has been confirmed in Figure 2. On the other hand, more water content causes a larger initial distance between the precursors of the polycondensation reaction, and the resulting reaction product is not enough to fill the gap between the particles themselves [52,53]. Moreover, excessive water is not consumed in the geopolymerization. As the reaction proceeds, water evaporates and leaves pores in the system [21]. The above two effects offset each other, and the influence of water content is dominant. Therefore, although the increase in L/S ratio can increase the reaction extent and the number of products, the pore becomes larger; Chotetanorm et al. [54] also found the same rules.

### 3.5. Morphology Analysis

**Figure 7:**
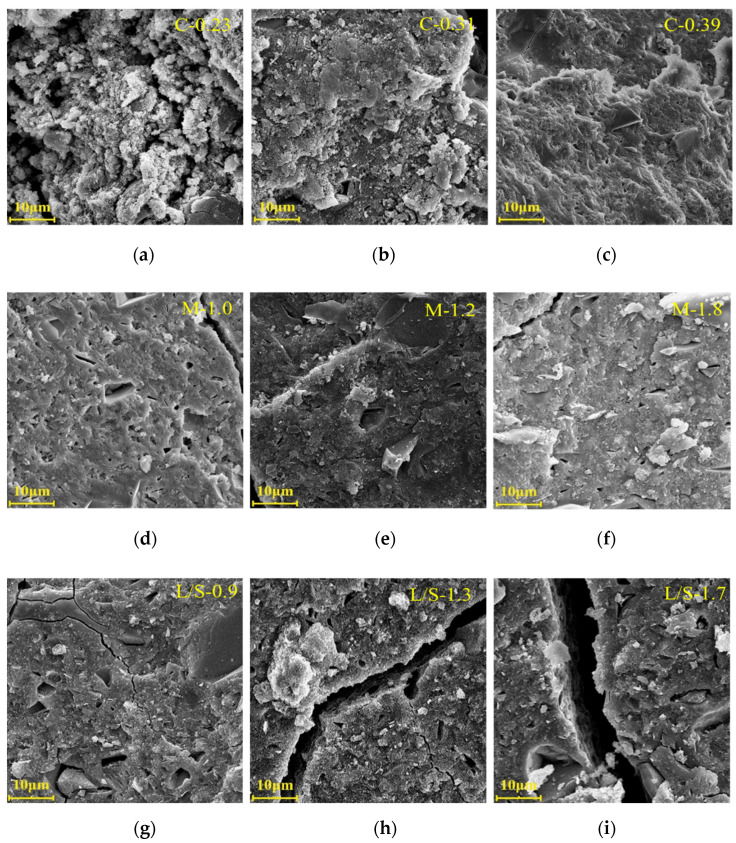
SEM images of MK/GGBFS-blended geopolymer pastes synthesized with varying concentrations (**a**)–(**c**), moduli of alkaline activator (**d**)–(**f**), and L/S ratio (**g**)–(**i**).

Figure 7 shows the SEM image of the MK/GGBFS-blended geopolymer pastes synthesized with varying concentrations, moduli of the alkaline activator, and L/S ratios, and all samples have unreacted mineral raw material particles and products with different morphologies. It can be seen from Figure 7a–c that the microstructure of the sample with the lowest concentration (C-0.23) is loose, and there are large interconnected pores between the gel phase formed by the reaction and the unreacted mineral raw material particles. In contrast, the gel phase in the microstructure of sample C-0.39 is the densest, which has small and disconnected pores and less unreacted mineral raw material particles; this is consistent with the previous results that the higher the concentration, the smaller the porosity of geopolymer paste, the finer the pore size, and the higher the reaction extent. This is because with the increase in concentration, the alkali content and active SiO_2_ content in the system increase, and the strong alkali can promote the depolymerization of the mineral raw materials, while the increase in active SiO_2_ content promotes the formation of gel products and continuously fills the pores. The change of the modulus has the least effect on the microstructure of the samples (Figure 7d–f) because the samples M-1.0, M-1.2, and M-1.8 all have a high reaction extent (more than 65%). An increase in the L/S ratio favored the formation of cracks (Figure 7g–i). The increase in the L/S ratio improves the reaction extent and the total porosity at the same time. The water consumed in the dissolution stage is released in the form of products in the polycondensation stage and filled between the primary particles or secondary particles of the gel. After the paste is hardened, the water gradually evaporates, leaving pores and cracks, affecting the degree of polymerization of the product, thus deteriorating the microstructure of the paste. Hence, to ensure workability, the lowest L/S ratio is recommended to formulate geopolymer pastes.

### 3.6. Setting Time and Compressive Strength

Table 5 lists the setting time and compressive strength of the MK/GGBFS-blended geopolymer pastes synthesized with varying concentrations, moduli of the alkaline activator, and L/S ratios. With an increase in the concentration and modulus of the alkaline activator, both the initial and final setting times decreased, whereas the setting times increased with an increase in the L/S ratio. These results agree well with the results of isothermal calorimetry. The high concentration and modulus of the alkaline activator accelerated the geopolymeric reaction and thus the setting. Conversely, a high L/S ratio lowered the geopolymeric reaction and thus the setting.

In a previous study, the setting times increased with an increase in the modulus in an MK/GGBFS-blended system [35], in contrast to the current study. This may be attributed to the different concentrations in both systems. A low concentration is insufficient or slow to dissolve the precursors into alumina monomers, which plays a critical role in network formation. The geopolymeric reaction does not occur without alumina, even if substantial amounts of silica are available [55]. 

The alkaline activator used in reference [35] may always be in a state of low alkali content, and the increase in modulus further reduces the alkali concentration in the system. Therefore, the alkali-activated MK/GGBFS-blended system in this study requires more time to achieve the required number of aluminum monomers when the geopolymerization occurs. However, in our study, the alkaline activator used has always been able to depolymerize the mineral raw materials to form a sufficient number of active silicon and aluminum monomers. At the same time, the increase in modulus provides more silicate for the geopolymerization. Therefore, the setting time of the paste is shortened with the increase in the modulus.

This perspective can be verified by another study [56], in which the setting times were shortened with an increase in modulus from 0 to 1.5, and then lengthened with an increase in modulus from 1.5 to 2.0. This is because the alkali content was sufficient to rapidly dissolve the raw materials when the modulus was lower than 1.5 and insufficient when the modulus was higher than 1.5.

**Figure 8:**
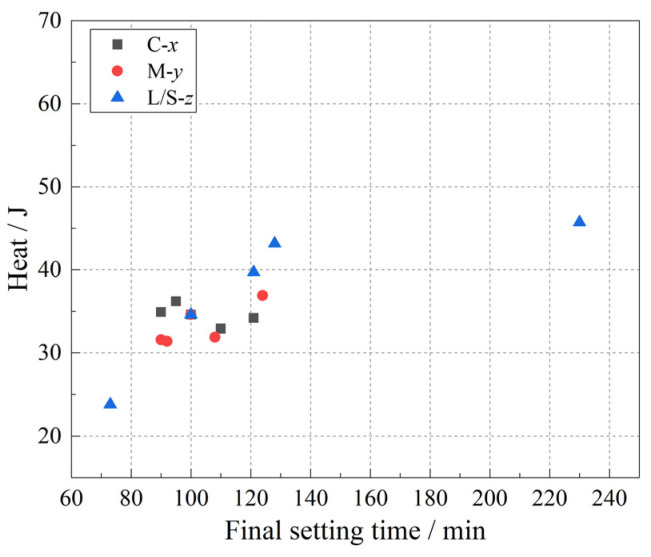
Heat released as a function of final setting time.

Figure 8 shows the heat released as a function of the final setting time. In the concentration and modulus variables, the final setting times corresponded to the heat released ranging from 30 to 40 J. However, with respect to the L/S ratio, the heat released corresponding to the final setting time occurred in a wide range from 23 to 46 J. This further shows that there is a high alkali content and active Si content in the alkaline activator selected in our experiment. Therefore, in the case of the same L/S ratio, the reaction extent required for the geopolymerization to achieve the final setting of the geopolymer fresh paste is not much different; however, the increase in L/S ratio leads to the increase in water content in the system, which reduces the heat released by the geopolymerization. Therefore, when the L/S ratio is changed, the reaction extent required for the system to achieve final condensation is different.

The compressive strength of the pastes increased with increasing alkaline activator concentration. When the modulus increased from 1.0 to 1.8, the compressive strength increased and reached a maximum of 49.4 MPa at a modulus of 1.2 and then decreased along with a continuous increase in modulus. The compressive strength decreased with increasing L/S ratio.

**Figure 9:**
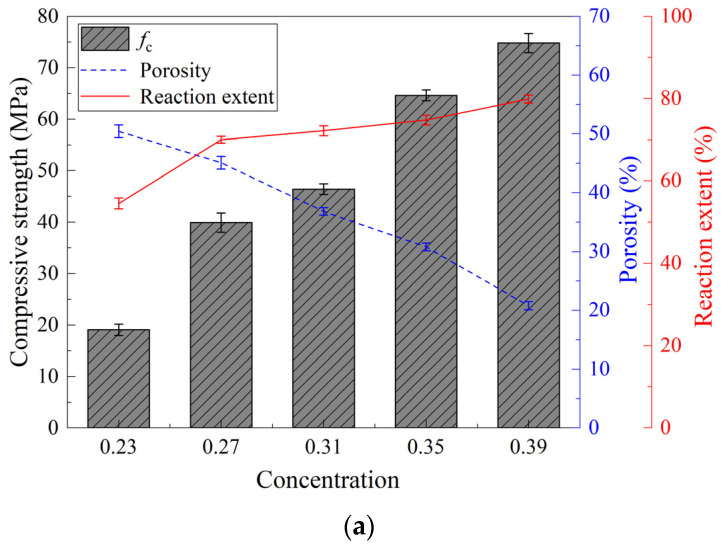

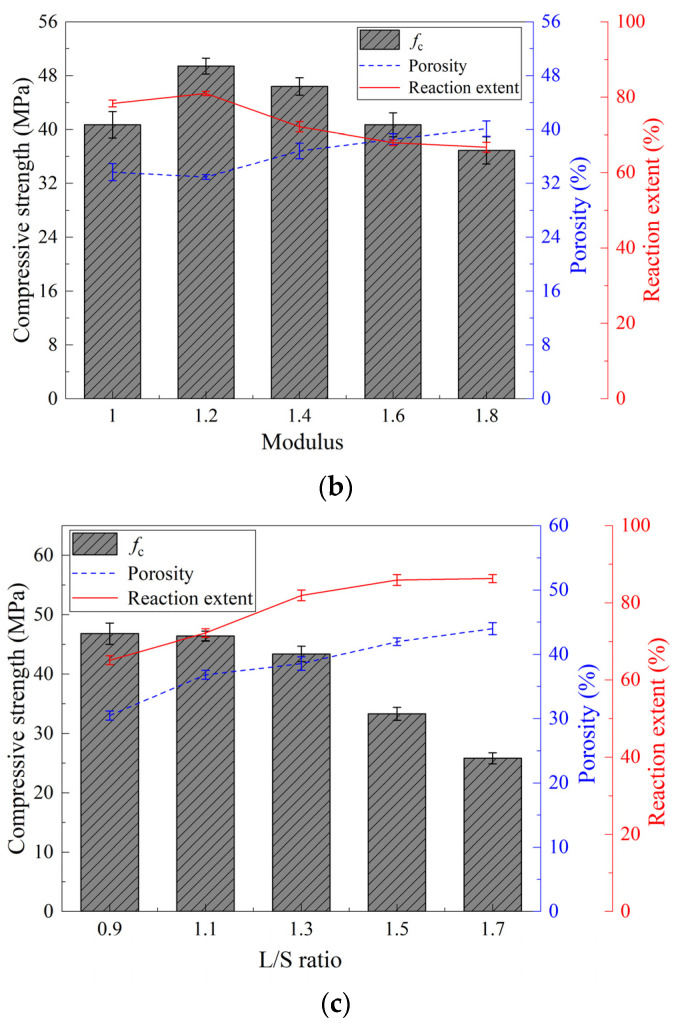
Compressive strength as a function of reaction extent and porosity: concentrations (**a**), moduli (**b**), and L/S ratios (**c**).

Figure 9 shows the compressive strength as a function of the reaction extent and porosity. For the concentration and modulus variables, the compressive strength is significantly correlated to the reaction extent, whereas it is inversely related to the reaction with respect to the L/S ratio. This can be attributed to the reaction extent and porosity concurred with the determination of compressive strength. For the variables of concentration and modulus, the reaction extent increased with concomitant porosity reduction, which had a synergetic effect on the compressive strength improvement. With respect to the L/S ratio, the negative effect of the increase in porosity contributing to the water content increase was significantly higher than the promotion effect of the reaction extent on the compressive strength.

As shown in Table 6, compared with the sample C-0.27, the sample L/S-1.7e has lower porosity and a higher reaction extent and contains more available silicate (higher concentration with the same modulus), which is expected to have a higher compressive strength while exhibiting a significantly lower compressive strength. This is because the increase in water content lowers the degree of polymerization because water participates in the geopolymer network [57]. 

## 4. Conclusions

In this study, the effects of the alkaline activator concentration, modulus, and the L/S ratio on the setting time and compressive strength of MK/GGBFS-blended geopolymer pastes were studied. The relationships between the reaction extent, total heat released, porosity, setting time, and compressive strength under different variables were confirmed. It was found that:

1. The exothermic characteristics of geopolymerization in the fresh geopolymer paste are related to the alkali content, silicate content, and water content in the alkaline activator. With the increase in concentration, the alkali content increased, the time of exothermic peak appeared earlier, the peak value increased, and the heat production increased. With the increase in modulus, the silicate content increases, the time of exothermic peak appears earlier, and the peak value increases, but the heat production increases first and then decreases, and reaches the highest value when the modulus is 1.2, which is related to the high alkali content that hinders the continuous geopolymerization. As the L/S ratio increases, the alkali content and silicate content increase, and the heat production increases. However, as the water content also increases, the time when the exothermic peak appears is delayed and the peak value decreases.
2. The change law of the reaction extent of geopolymer paste with the three influencing factors is consistent with the heat production of geopolymerization, but the fresh geopolymer paste with large heat production does not necessarily have a higher reaction extent, which is related to whether there is a difference in the amount of silicate in the paste. There is a maximum reaction extent of geopolymer paste, and the maximum value is 86.3% under the experimental conditions in this paper. The variation of porosity and the most probable pore size with the three influencing factors is opposite to the heat production. The difference of the total porosity caused by the different influencing factors is mainly related to the porosity with pore size less than or equal to 30 nm.
3. The initial setting time of geopolymer paste mainly depends on the time and peak value of the exothermic peak of the geopolymerization. The earlier the exothermic peak appears, the greater the peak value, the faster the rate of geopolymerization, the more intense the degree, the shorter the initial setting time of the geopolymer paste.
4. The compressive strength of the geopolymer paste increases with the increase in concentration. With the increase in modulus, the compressive strength increases first and then decreases, reaching a maximum of 49.4 MPa when the modulus is 1.2. The variation of compressive strength with concentration or modulus is the same as that of the reaction extent, which is opposite to the total porosity. The compressive strength decreases with the increase in the L/S ratio. When the L/S ratio changes, the compressive strength is positively correlated with the reaction extent and negatively correlated with total porosity. The total porosity and reaction extent jointly determine the compressive strength of the geopolymer paste.
5. In this paper, the reaction extent and the total porosity of the geopolymer paste jointly determine the compressive strength. In the future, further tests can be carried out to clarify when the reaction degree or porosity plays a leading role. The microstructure of the geopolymer paste with different mixing ratios was observed by SEM. In the future, EDS can be used to analyze the specific products in the paste.

## Figures and Tables

**Table 1:** Main chemical components of MK and GGBFS (wt.%).

Component	SiO_2_	Al_2_O_3_	CaO	MgO	SO_3_	TiO_2_	K_2_O	Fe_2_O_3_
MK	52.53	45.42	0.26	-	0.04	0.97	0.18	-
GGBFS	30.23	13.72	44.06	5.58	3.16	1.79	0.50	0.41

**Table 2:** Synthesis formulations of MK/GGBFS-blended geopolymer pastes.

No.	Concentration	Modulus	L/S	GGBFS (g)	MK (g)	Sodium Silicate Solution (g)	H_2_O (g)	NaOH (g)
C-0.23	0.23	1.4	1.1	40	60	55.1	47.0	7.9
C-0.27	0.27			40	60	64.7	36.0	9.3
C-0.31	0.31			40	60	74.3	25.1	10.7
C-0.35	0.35			40	60	83.8	14.1	12.0
C-0.39	0.39			40	60	93.4	3.1	13.4
M-1.0	0.31	1.0	1.1	40	60	63.5	31.0	15.5
M-1.2		1.2		40	60	69.4	27.8	12.9
M-1.4		1.4		40	60	74.3	25.1	10.7
M-1.6		1.6		40	60	78.4	22.8	8.8
M-1.8		1.8		40	60	82.0	20.8	7.2
L/S-0.9	0.31	1.4	0.9	40	60	60.8	20.5	8.7
L/S-1.1			1.1	40	60	74.3	25.1	10.7
L/S-1.3			1.3	40	60	87.8	29.6	12.6
L/S-1.5			1.5	40	60	101.2	34.1	14.5
L/S-1.7			1.7	40	60	114.8	38.7	16.5

**Table 3:** Total heat released during the first 72 h.

Code	Total Heat Released (J)			Average	Standard Deviation
C-0.23	179.3	176.2	168.6	174.7	5.51
C-0.27	196.0	193.0	184.0	191.0	6.24
C-0.31	235.0	228.6	224.3	229.3	5.38
C-0.35	260.0	257.4	247.9	255.1	6.37
C-0.39	290.5	287.2	280.3	286.0	5.20
M-1.0	179.3	176.2	168.6	243.0	6.51
M-1.2	196.0	193.0	184.0	259.0	5.99
M-1.4	235.0	228.6	224.3	229.3	6.38
M-1.6	260.0	257.4	247.9	209.5	6.41
M-1.8	290.5	287.2	280.3	181.5	3.63
L/S-0.9	216.2	210.8	209.9	212.3	3.41
L/S-1.1	234.6	227.2	226.1	229.3	4.62
L/S-1.3	238.5	232.6	232.1	234.4	3.56
L/S-1.5	256.3	248.3	249.6	251.4	4.29
L/S-1.7	273.5	265.8	267.7	269.0	4.01

**Table 4:** The porosity of the different pore sizes and the most probable pore sizes.

No.	The Most Probable Pore Sizes/nm	Total Porosity/%	The Porosity of the Different Pore Sizes/%			
			<10 nm	10–100 nm	100–1000 nm	>1000 nm
C-0.23	102.6	50.4	4.9	23.3	20.6	1.6
C-0.27	26.0	45.1	3.4	39.4	0.6	1.7
C-0.31	15.7	36.8	3.0	33.2	0.4	0.2
C-0.35	12.1	30.8	5.5	23.9	0.2	1.2
C-0.39	8.6	20.8	10.8	7.6	0.7	1.7
M-1.0	13.5	33.7	3.9	27.5	0.4	1.9
M-1.2	11.4	32.9	6.3	24.0	0.6	2.0
M-1.4	15.7	36.8	3.0	33.2	0.4	0.2
M-1.6	17.0	38.6	2.5	34.4	0.4	1.3
M-1.8	24.4	40.1	2.2	36.5	0.4	1.0
L/S-0.9	13.4	30.5	3.8	25.7	0.2	0.8
L/S-1.1	15.7	36.8	3.0	33.2	0.4	0.2
L/S-1.3	16.9	38.6	2.8	33.1	0.3	2.4
L/S-1.5	18.1	42.0	2.2	37.6	0.3	1.9
L/S-1.7	18.7	44.0	2.2	40.4	0.2	1.2

**Table 5:** Setting time and compressive strength of MK/GGBFS-blended geopolymer pastes.

No.	*T*_i_ ^1^(min)	*T*_f_ ^2^(min)	28d *f*_c_ ^3^(MPa)
C-0.23	116	121	19.1
C-0.27	106	110	39.9
C-0.31	91	100	46.4
C-0.35	89	95	64.6
C-0.39	85	90	74.8
M-1.0	120	124	40.7
M-1.2	104	108	49.4
M-1.4	91	100	46.4
M-1.6	88	92	40.7
M-1.8	85	90	36.9
L/S-0.9	68	73	46.8
L/S-1.1	91	100	46.4
L/S-1.3	115	121	43.4
L/S-1.5	118	128	33.3
L/S-1.7	226	230	25.8

^1^ T_i_ denotes the initial setting time of the samples. ^2^ T_f_ denotes the final setting time of the samples. ^3^ 28d *f*_c_ denotes the 28-day compressive strength of the samples.

**Table 6:** Comparison of porosity, reaction extent, and compressive strength between samples L/S-1.7 and C-0.27.

Code	L/S-1.7 (C-0.31, M-1.2)	C-0.27 (L/S-1.1, M-1.2)
Porosity (%)	43.97	45.08
Reaction extent (%)	86.31	70.01
Compressive strength (MPa)	25.08	39.90

## Data Availability

Not applicable.
